# Task demands affect spatial reference frame weighting during tactile localization in sighted and congenitally blind adults

**DOI:** 10.1371/journal.pone.0189067

**Published:** 2017-12-11

**Authors:** Jonathan T. W. Schubert, Stephanie Badde, Brigitte Röder, Tobias Heed

**Affiliations:** 1 Biological Psychology and Neuropsychology, Faculty of Psychology and Human Movement Science, University of Hamburg, Hamburg, Germany; 2 Department of Psychology, New York University, New York, United States of America; 3 Biopsychology & Cognitive Neuroscience, Faculty of Psychology & Sports Science, Bielefeld University, Bielefeld, Germany; 4 Center of Excellence in Cognitive Interaction Technology (CITEC), Bielefeld University, Bielefeld, Germany; Pennsylvania State University, UNITED STATES

## Abstract

Task demands modulate tactile localization in sighted humans, presumably through weight adjustments in the spatial integration of anatomical, skin-based, and external, posture-based information. In contrast, previous studies have suggested that congenitally blind humans, by default, refrain from automatic spatial integration and localize touch using only skin-based information. Here, sighted and congenitally blind participants localized tactile targets on the palm or back of one hand, while ignoring simultaneous tactile distractors at congruent or incongruent locations on the other hand. We probed the interplay of anatomical and external location codes for spatial congruency effects by varying hand posture: the palms either both faced down, or one faced down and one up. In the latter posture, externally congruent target and distractor locations were anatomically incongruent and vice versa. Target locations had to be reported either anatomically (“palm” or “back” of the hand), or externally (“up” or “down” in space). Under anatomical instructions, performance was more accurate for anatomically congruent than incongruent target-distractor pairs. In contrast, under external instructions, performance was more accurate for externally congruent than incongruent pairs. These modulations were evident in sighted and blind individuals. Notably, distractor effects were overall far smaller in blind than in sighted participants, despite comparable target-distractor identification performance. Thus, the absence of developmental vision seems to be associated with an increased ability to focus tactile attention towards a non-spatially defined target. Nevertheless, that blind individuals exhibited effects of hand posture and task instructions in their congruency effects suggests that, like the sighted, they automatically integrate anatomical and external information during tactile localization. Moreover, spatial integration in tactile processing is, thus, flexibly adapted by top-down information—here, task instruction—even in the absence of developmental vision.

## Introduction

The brain continuously integrates information from multiple sensory channels [1–6]. Tactile localization, too, involves the integration of several information sources, such as somatosensory information about the stimulus location on the skin with proprioceptive and visual information about the current body posture, and has therefore been investigated in the context of information integration within and across the senses. We have suggested that tactile localization involves at least two cortical processing steps [7,8]. When tactile information first arrives in the cortex, it is initially encoded relative to the skin in an anatomical reference frame, reflected in the homuncular organization of the somatosensory cortex [9]. This information is consecutively remapped into an external reference frame. By merging anatomical skin-based spatial information with proprioceptive, visual, and vestibular signals, the brain derives an external spatial location, a process referred to as tactile remapping [10–14]. The term ‚external’, in this context, denotes a spatial code that abstracts from the original location, but is nevertheless egocentric, and takes into account the spatial relation of the stimulus to the eye and body [12,15]. In a second step, information coded with respect to the different reference frames is integrated, presumably to derive a superior tactile location estimate [16]. For sighted individuals, this integration of different tactile codes appears mandatory [17–19]. Yet, the relative weight of each code is subject to change depending on current task demands: external spatial information is weighted more strongly when task instructions emphasize external spatial aspects [7,8,16], in the context of movement [20–26], and in the context of frequent posture changes [27]. Thus, the tactile localization estimate depends on flexibly weighted integration of spatial reference frames.

However, the principles that guide integration for tactile localization critically depend on visual input after birth. Differences between sighted and congenitally blind participants in touch localization are evident, for instance, in tasks involving hand crossing. Hand crossing over the midline allows to experimentally misalign anatomical and external spatial reference frames, so that the left hand occupies the right external space and vice versa. This posture manipulation reportedly impairs tactile localization compared to an uncrossed posture in sighted, but not in congenitally blind individuals [28,29]. Similarly, hand crossing can attenuate spatial attention effects on somatosensory event-related potentials (ERP) between approximately 100 and 250 ms post-stimulus in sighted, but not in congenitally blind individuals [30]. Together, these previous studies indicate that congenitally blind individuals, unlike blind individuals, may not integrate externally coded information with anatomical skin-based information by default when they process touch.

Recent studies, however, have cast doubt on this conclusion. For instance, congenitally blind individuals used external along with anatomical coding when tactile stimuli had to be localized while making bimanual movements [22]. Evidence for automatic integration of external spatial information in congenitally blind individuals comes not only from tactile localization, but, in addition, from a bimanual coordination task: when participants moved their fingers symmetrically, this symmetry was encoded relative to external space rather than according to anatomical parameters such as the involved muscles, similar as in sighted participants [31]. In addition, it has recently been reported that early blind individuals encode locations of motor sequences relative to both external and anatomical locations [32]. Moreover, early blind individuals appear to encode time relative to external space, and this coding strategy may be related to left-right finger movements during Braille reading [33]. These studies suggest that congenitally blind humans, too, integrate spatial information coded in different reference frames according to a weighting scheme [16], but may use lower default weights for externally coded information than sighted individuals. In both groups, movement contexts seem to induce stronger weighting of external spatial information.

In sighted individuals, task demands are a second factor besides movement context that can modulate the weighting of spatial information in tactile localization. For instance, tactile temporal order judgments (TOJ), that is, the decision which of two tactile locations was stimulated first, are sensitive to the conflict between anatomical and external locations that arises when stimuli are applied to crossed hands [18,19]. This crossing effect was modulated by a secondary task that accentuated anatomical versus external space, indicating that the two tactile codes were weighted according to the task context [8]. A modulatory effect of task demands on tactile spatial processing in sighted individuals is also evident in the tactile congruency task: In this task, tactile distractors presented to one hand interfere with elevation judgements about simultaneously presented tactile target stimuli presented at the other hand [34,35]. In such tasks, one can define spatial congruency between target and distractor in two ways: In an anatomical reference frame two stimuli are congruent if they occur at corresponding skin locations; in an external reference frame two stimuli are congruent if they occur at corresponding elevations. If one places the two hands in the same orientation, for instance, with both palms facing down, anatomical and external congruency are in correspondence. However, when the palm of one hand faces up and the other down, two tactile stimuli presented at upper locations in external space will be located at incongruent anatomical skin locations, namely at the palm of one, and at the back of the other hand. Thus, anatomical and external congruency do not correspond in this latter posture. A comparison of congruency effects between these postures provides a measure of the weighting of anatomical and external tactile codes. Whether congruency effects in this task were encoded relative to anatomical or relative to external space was modifiable by both task instructions and response modalities in a sighted sample [34]. This modulation suggests that the weighting of anatomical and external spatial information in the tactile congruency task was flexible, and was modulated by task requirements.

With respect to congenitally blind humans, evidence as to whether task instructions modulate spatial integration in a similar way as in sighted individuals is currently indirect. Effects of task context have been suggested to affect reaching behavior of blind children [36]. For tactile integration, one piece of evidence in favor of an influence of task instructions comes from the comparison of two very similar studies that have investigated tactile localization in early [37] and congenitally blind humans [30] by comparing somatosensory ERPs elicited by tactile stimulation in different hand postures. Both studies asked participants to report infrequent tactile target stimuli on a pre-cued hand, but observed contradicting results: One study reported an attenuation of spatial attention-related somatosensory ERPs between 140 and 300 ms post-stimulus to non-target stimuli with crossed compared to uncrossed hands [37], suggesting that external location had affected tactile spatial processing in early blind participants. The other study [30], in contrast, did not observe any significant modulation of spatial attention-related somatosensory ERPs by hand posture and concluded that congenitally blind humans do not, by default, use external spatial information for tactile localization. The two studies differed in how participants were instructed about the to-be-monitored location. In the first study, the pitch of an auditory cue indicated the task-relevant side relative to external space in each trial [37]. In the second study, in contrast, the pitch of a cuing sound referred to the task-relevant hand, independent of hand location in external space [30]. One possible explanation for the observed differences in ERP effects across studies, thus, is that task instructions modulate how anatomical and external information is weighted in congenitally blind individuals as they do in the sighted.

Here, we investigated the weighting of anatomical and external reference frames by means of an adapted version of the tactile congruency task [34,35]. Sighted and congenitally blind participants localized vibro-tactile target stimuli, presented randomly on the palm or back of one hand, while ignoring vibro-tactile distractors on the palm or back of the other hand. Thus, distractors could appear at an anatomically congruent or incongruent location. Hand posture varied, either with both palms facing down, or with one palm facing down and the other up. With differently oriented hands, anatomically congruent stimuli were incongruent in external space and vice versa. We used this experimental manipulation to investigate the relative importance of anatomical and external spatial codes during tactile localization.

We introduced two experimental manipulations to investigate the role of task demands on the weighting of anatomical and external spatial information: a change of task instructions and a change of the movement context.

For the manipulation of task instructions, every participant performed two experimental sessions. In one session, responses were instructed anatomically, that is, with respect to palm or back of the hand. In a second session, responses were instructed externally, that is, with respect to upper and lower locations in space. We hypothesized that each task instruction would emphasize the weighting of the corresponding reference frame. This means that with differently oriented hands, that is, when anatomical and external reference frames are misaligned, the direction of the congruency effect should depend on task instructions.

With the manipulation of movement context, we aimed at corroborating previous results suggesting that movement planning and execution as well as frequent posture changes lead to emphasized weighting of external spatial information [27]. Accordingly, we hypothesized that frequent posture changes would increase the weight of the external reference frame in a similar way in the present task. To this end, participants either held their hands in a fixed posture for an entire experimental block, or they changed their hand posture in a trial-by-trial fashion. Again, with differently oriented hands, changes in the weighting of anatomical and external spatial information would be evident in a modulation of tactile congruency effects. If frequent posture changes, compared to a blockwise posture change, induce an increased weighting of external information, this will result in a decrease of anatomical congruency effects under anatomical instructions and in an increase of external congruency effects under external instructions.

## 1. Methods

We follow open science policies as suggested by the Open Science Framework (see https://osf.io/hadz3/wiki/home/) and report how we determined the sample size, all experimental manipulations, all exclusions of data, and all evaluated measures of the study. Data and analysis scripts are available online (see https://osf.io/ykqhd/). For readability, we focus on accuracy in the paper, but report reaction times and their statistical analysis in the supporting information (Methods A, Results A-E, Figs A-D, and Tables A,B in S1 File). Previous studies have reported qualitatively similar results for the two measures (e.g. [38]), and results were also comparable in the present study.

### 1.1. Participants

The size of our experimental groups was constrained by the availability of congenitally blind volunteers; we invited every suitable participant we identified within a period of 6 months. Group size is comparable to that of previous studies that have investigated spatial coding in the context of tactile congruency. We report data from sixteen congenitally blind participants (8 female, 15 right handed, 1 ambidextrous, age: M = 37 years, SD = 11.6, range: 19 to 53) and from a matched control group of sixteen blindfolded sighted participants (8 female, all right handed, age: M = 36 years, SD = 11.5, range: 19 to 51). All sighted participants had normal or corrected-to-normal vision. Blind participants were visually deprived since birth due to anomalies in peripheral structures resulting either in total congenital blindness (n = 6) or in minimal residual light perception (n = 10). Peripheral defects included binocular anophthalmia (n = 1), retinopathy of prematurity (n = 4), Leber’s congenital amaurosis (n = 1), congenital optical nerve atrophy (n = 2), and genetic defects that were not further specified (n = 8). All participants gave informed written consent and received course credit or monetary compensation for their participation. The study was approved by the ethical board of the German Psychological Society (TB 122010) and conducted in accordance with the standards laid down in the Declaration of Helsinki.

Of twenty originally tested congenitally blind participants, one did not complete the experiment, and data from three participants were excluded due to performance at chance level (see below). We recruited 45 sighted participants to establish a group of 16 control participants. Technical failure during data acquisition prevented the use of data from two out of these 45 participants. Furthermore, we had developed and tested the task in a young, sighted student population. We then tested the blind participants before recruiting matched controls from the population of Hamburg via online and newspaper advertisement. For many sighted, age-matched participants older than 30 years, reacting to the target in the presence of an incongruent distractor stimulus proved too difficult, resulting in localization performance near chance level in the tactile congruency task. Accordingly, 23 sighted participants either decided to quit, or were not invited for the second experimental session because their performance in the first session was not sufficient. We address this surprising difference between blind and sighted participants in ability to perform the experimental task in the Discussion.

### 1.2. Apparatus

Participants sat in a chair with a small lap table on their legs. They placed their hands in a parallel posture in front of them, with either both palms facing down (termed "same orientation") or with one hand flipped palm down and the other palm up (termed "different orientation"). Whether the left or the right hand was flipped in the other orientation condition was counterbalanced across participants. Distance between index fingers of the hands was approximately 20 cm, measured while holding both palms down.

**Fig 1:**
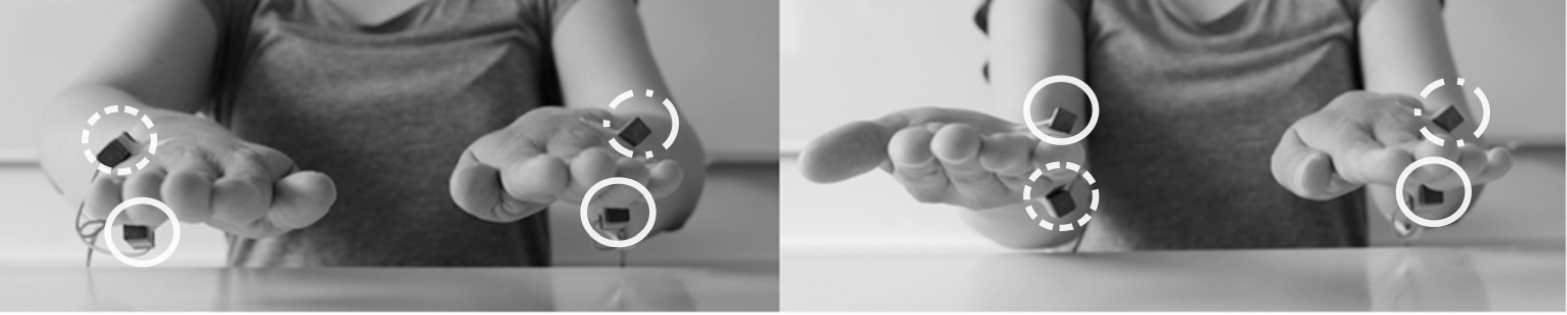
Experimental setup. Four vibro-tactile stimulators were attached to the palm and back of each hand (marked with white circles). The hands were either held in the same orientation with both palms facing downwards (**A**) or in different orientations with one hand flipped upside-down (**B**). In each trial, a target stimulus was randomly presented at one of the four locations. Simultaneously, a distractor stimulus was presented randomly at one of the two stimulator locations on the other hand. Target and distractor stimuli differed with respect to their vibration pattern. Participants were asked to localize the target stimulus as quickly and accurately as possible. For statistical analysis and figures, stimulus pairs presented to the same anatomical locations were defined as congruent, as illustrated by dashed (target) and dashed-dotted (distractor) circles, which both point to the back of the hand here. Note that with differently oriented hands (**B**) anatomically congruent locations are incongruent in external space and vice versa.

Foam cubes supported the hands for comfort, and to avoid that stimulators touched the table. Custom-built vibro-tactile stimulators were attached to the back and to the palm of both hands midway between the root of the little finger and the wrist (Fig 1). Participants wore earplugs and heard white noise via headphones to mask any sound produced by the stimulators. We monitored hand posture with a movement tracking system (Visualeyez II VZ4000v PTI; Phoenix Technologies Incorporated, Burnaby, Canada), with LED markers attached to the palm and back of the hands. We controlled the experiment with Presentation (version 16.2; Neurobehavioral Systems, Albany, CA, USA), which interfaced with Matlab (The Mathworks, Natick, MA, USA) and tracker control software VZSoft (PhoeniX Technologies Incorporated, Burnaby, Canada).

### 1.3. Stimuli

The experiment comprised two kinds of tactile stimuli: targets, to which participants had to respond, and distractors, which participants had to ignore. Stimuli were equally distributed both with respect to which hand and which location, up or down, was stimulated. The distractor always occurred on the other hand than the target, at an anatomically congruent location in half of the trials, and at an anatomically incongruent location in the other half of the trials (cf. Fig 1). Target stimuli consisted of 200 Hz stimulation for 235 ms. Distractor stimuli vibrated with the same frequency, but included two gaps of 65 ms, resulting in three short bursts of 35 ms each. We initially suspected that our sighted control participants' low performance in the congruency task may be related to difficulty in discriminating target and distractor stimuli. Therefore, we adjusted the distractor stimulus pattern for the last seven recruited control participants if they could not perform localization of the target above chance level in the presence of an incongruent distractor stimulus during a pre-experimental screening; such adjustments were, however, necessary for only three of these last seven participants, for the four other participants no adjustments were made. In a first step, we increased the distractor's gap length to 75 ms, resulting in shorter bursts of 25 ms (1 participant). If the participant still performed at chance level in incongruent trials of the localization task, we set the distractor pattern to 50 ms “on”, 100 ms “off”, 5 ms “on”, 45 ms “off”, and 35 ms “on” (2 participants). Note that, while these distractor stimulus adjustments made discrimination between target and distractor easier, they did not affect target localization per se.

It is possible that, for participants undergoing stimulus adjustment, localization performance in incongruent trials simply improved due to the additional training. Importantly, however, stimuli were the same in all experimental conditions. Yet, to ascertain that statistical results were not driven by the three respective control participants, we ran all analyses both with and without their data. The overall result pattern was unaffected, and we report results of the full control group. In addition, we ascertained that both sighted and blind groups could discriminate target and distractor stimuli equally well. Practice before the task included a block of stimulus discrimination; performance did not differ significantly between the two groups in this phase of the experiment (Results F in S1 File). Moreover, discrimination performance during the practice block and the size of congruency effects did not significantly correlate (Results F in S1 File).

### 1.4. Procedure

The experiment was divided into four large parts according to the combination of the two experimental factors Instruction (anatomical, external) and Movement Context (static vs. dynamic context, that is, blockwise vs. frequent posture changes). The order of these four conditions was counterbalanced across participants. Participants completed both Movement Context conditions under the first instruction within one session, and under the second instruction in another session, which took part on another day. Participants completed four blocks of 48 trials for each combination of Instruction and Movement Context. Trials in which participants responded too fast (RT < 100 ms), or not at all, were repeated at the end of the block (6.35% of trials).

### 1.5. Manipulation of instruction

Under external instructions, participants had to report whether the target stimulus was located “up” or “down” in external space and ignore the distractor stimulus. They had to respond as fast and accurately as possible by means of a foot pedal placed underneath one foot (left and right counterbalanced across participants), with "up" responses assigned to lifting of the toes, and "down" responses to lifting of the heel. Previous research in our lab showed that participants strongly prefer this response assignment and we did not use the reverse assignment to prevent increased task difficulty. Note, that congruent and incongruent target-distractor combinations required up and down responses with equal probability, so that the response assignment did not bias our results. Under anatomical instructions, participants responded whether the stimulus had occurred to the palm or back of the hand. We did not expect a preferred response mapping under these instructions and, therefore, balanced the response mapping of anatomical stimulus location to toe or heel across participants.

### 1.6. Manipulation of movement context

Under each set of instructions, participants performed the entire task once with a constant hand posture during entire experimental blocks (static movement context), and once with hand posture varying from trial to trial (dynamic movement context).

#### 1.6.1. Static movement context

In the static context, posture was instructed verbally at the beginning of each block. A tone (1000 Hz sine, 100 ms) presented via loudspeakers placed approximately 1 m behind the participants signaled the beginning of a trial. After 1520–1700 ms (uniform distribution) a tactile target stimulus was presented randomly at one of the four locations. Simultaneously, a tactile distractor stimulus was presented at one of the two locations on the other hand. Hand posture was changed after completion of the second of four blocks.

#### 1.6.2. Dynamic movement context

In the dynamic context, an auditory cue at the beginning of each trial instructed participants either to retain (one beep, 1000 Hz sine, 100 ms) or to change (two beeps, 900 Hz sine, 100 ms each) the posture of the left or right hand (constant hand throughout the experiment, but counterbalanced across participants). After this onset cue, the trial continued only when the corresponding motion tracking markers attached to the hand surfaces had been continuously visible from above for 500 ms. If markers were not visible 5000 ms after cue onset, the trial was aborted and repeated at the end of the block. An error sound reminded the participant to adopt the correct posture. Tactile targets occurred equally often at each hand, so that targets and distracters, respectively, occurred half of the time on the moved, and half of the time on the unmoved hand. The order of trials in which posture changed and trials in which posture remained unchanged, was pseudo-randomized in a way to assure equal amounts of trials for both conditions.

### 1.7. Practice

Before data acquisition, participants familiarized themselves with the stimuli by completing two blocks of 24 trials in which each trial randomly contained either a target or a distractor, and participants reported with the footpedal which of the two had been presented. Next, participants localized 24 target stimuli without the presence of a distractor stimulus to practice the current stimulus-response mapping (anatomical instructions: palm and back of the hand vs. external instructions: upper or lower position in space to toes and heel). Finally, participants practiced five blocks of 18 regular trials, two with the hands in the same orientation, and three with the hands in different orientations. Auditory feedback was provided following incorrect responses during practice, but not during the subsequent experiment.

### 1.8. Data analysis

Data were analyzed and visualized in R (R Core Team, 2017) using the R packages lme4 (v1.1–9) [39], afex (v0.14.2) [40], lsmeans (v2.20–2) [41], dplyr (v0.4.3) [42], and ggplot2 (v1.0.1) [43]. We removed trials when their reaction time was longer than the participant’s mean reaction time plus two standard deviations, calculated separately for each condition (4.94% of trials), or when reaction time exceeded 2000 ms (0.64% of trials).

We analyzed accuracy using generalized linear mixed models (GLMM) with a binomial link function[44,45]. It has been suggested that a full random effects structure should be used for significance testing in (G)LMM [46]. However, it has been shown that conclusions about fixed effect predictors do not diverge between models with maximal and models with parsimonious random effects structure [47], and it is common practice to include the maximum possible number of random factors when a full structure does not result in model convergence (e.g. [45,48]). In the present study, models reliably converged when we included random intercepts and random slopes for each main effect, but not for interactions. Significance of fixed effects was assessed with likelihood ratio tests comparing the model with the maximal fixed effects structure and a model that excluded the fixed effect of interest [49]. These comparisons were calculated using the afex package [40], and employed Type III sums of squares and sum-to-zero contrasts. Fixed effects were considered significant at p < 0.05. Post-hoc comparisons of significant interactions were conducted using approximate z-tests on the estimated least square means (lsmeans package [41]). The resulting p-values were corrected for multiple comparisons following the procedure proposed by Holm [50]. To assess whether the overall result pattern differed between groups, we fitted a GLMM with the fixed between-subject factor Group (sighted, blind) and fixed within-subjects factors Instruction (anatomic, external), Posture (same, different), Congruency (congruent, incongruent), and Movement Context (static, dynamic). Congruency was defined relative to anatomical locations for statistical analysis and figures. Subsequently, to reduce GLMM complexity and to ease interpretability, we conducted separate analyses for each group including the same within-subject fixed effects.

## 2. Results

We assessed how task instructions and movement context modulate the weighting of anatomically and externally coded spatial information in a tactile-spatial congruency task performed by sighted and congenitally blind individuals.

We report here on accuracy, but, in addition, present reaction times and their statistical analysis in the supporting information (Methods A, Results A-E, Figures A-D, and Tables A,B in S1 File). Overall, reaction times yielded a result pattern comparable to that of accuracy. Weight changes should become evident in a modulation of congruency effects for hand postures that induce misalignment between these different reference frames. With differently oriented hands, stimulus pairs presented to anatomically congruent locations are incongruent in external space and vice versa, whereas the two coding schemes agree when the hands are in the same orientation. Thus, a modulation of reference frame weighting by task instructions would be evident in an interaction of Instruction, Posture, and Congruency. Furthermore, a modulation of weights by the movement context would be evident in an interaction of Movement Context, Posture, and Congruency.

**Fig 2:**
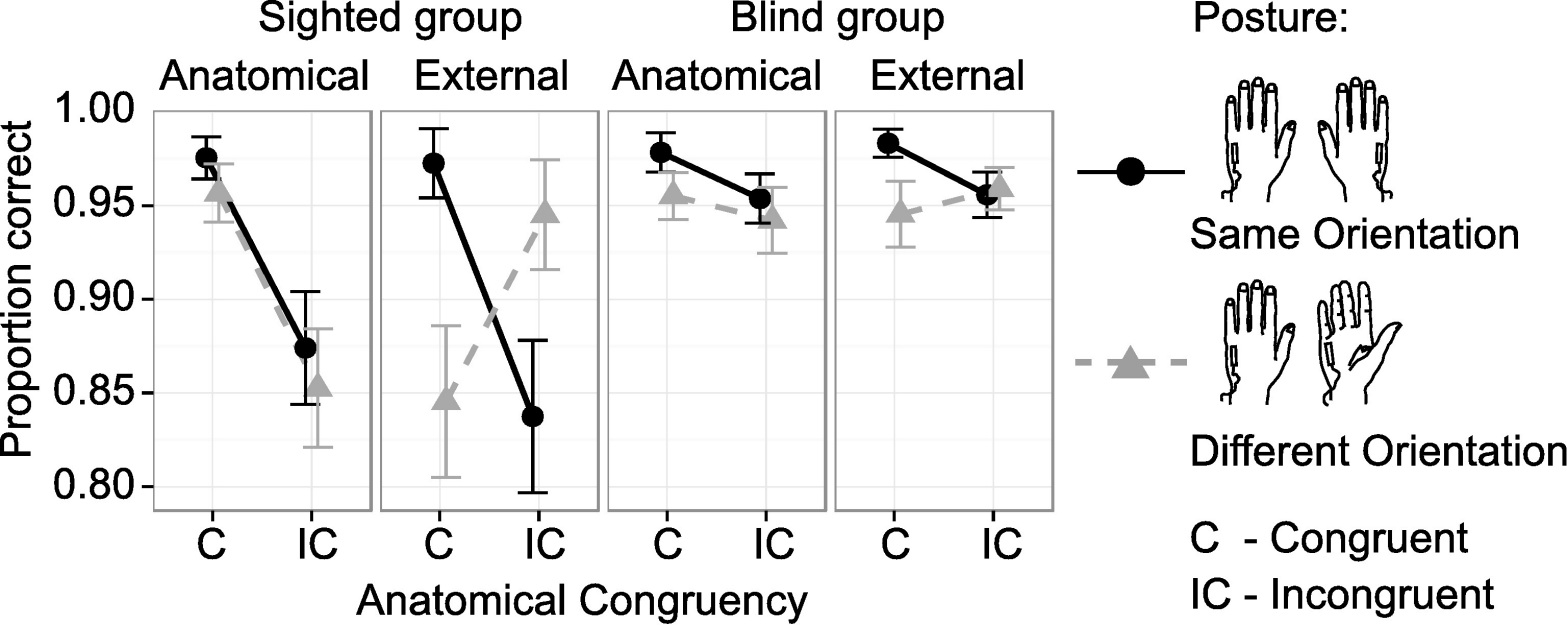
Accuracy in the tactile congruency task for factors Group, Instruction, Hand Posture, and Congruency (coded anatomically). Data are collapsed over static and dynamic movement conditions, as this manipulation did not render any significant results (see main text for details). Sighted (1^st^ and 2^nd^ column) and congenitally blind participants (3^rd^ and 4^th^ column) were instructed to localize tactile targets either relative to their anatomical (1^st^ and 3^rd^ column) or relative to their external spatial location (2^nd^ and 4^th^ column). Hands were placed in the same (black circles) and in different orientations (grey triangles). Tactile distractors were presented to anatomically congruent (C) and incongruent (IC) locations of the other hand and had to be ignored. Congruency is defined in anatomical terms (see Fig 1). Accordingly, with differently oriented hands, anatomically congruent stimulus pairs are incongruent in external space and vice versa. Whiskers represent the standard error of the mean. Note, that we present percentage-correct values to allow a comparison to previous studies (see methods for details), whereas for statistical analysis a log-linked GLMM was applied to single trials accuracy values.

We first conducted a GLMM that included all experimental factors, that is, Group, Instruction, Posture, Congruency, and Movement Context (see Table 1). Contrary to our hypotheses, this analysis did not reveal any relevant effects of the Movement Context on congruency effects (see Table 1 and Section 3.4; all p > 0.05). For readability, we therefore illustrate the experimental results in Fig 2 collapsed over the two Movement Context conditions (static vs. dynamic).

**Table 1:** Results of the full GLMM across both participant groups with accuracy as dependent variable.

Predictor	Estimate	SE	*χ*^2^	*p*
Intercept)	3.313	0.168		
Group	-0.282	0.168	2.69	0.101
Instruction	0.000	0.078	0.00	0.998
**Posture**	**-0.211**	**0.038**	**20.48**	**<0.001**
**Congruency**	**0.352**	**0.040**	**40.33**	**<0.001**
**Movement Context**	**-0.099**	**0.043**	**4.54**	**0.033**
Group X Instruction	0.034	0.078	0.19	0.662
Group X Posture	0.046	0.039	1.43	0.232
Instruction X Posture	0.027	0.034	0.63	0.428
**Group X Congruency**	**0.147**	**0.04**	**12.09**	**0.001**
**Instruction X Congruency**	**0.169**	**0.034**	**23.35**	**<0.001**
**Posture X Congruency**	**-0.365**	**0.034**	**120.5**	**<0.001**
*Group X Movement Context*	*0*.*080*	*0*.*043*	*3*.*47*	*0*.*063*
Instruction X Movement Context	-0.004	0.034	0.02	0.901
Posture X Movement Context	-0.038	0.034	1.20	0.273
Congruency X Movement Context	0.035	0.034	1.06	0.304
Group X Instruction X Posture	-0.047	0.034	1.85	0.174
**Group X Instruction X Congruency**	**0.145**	**0.034**	**17.57**	**<0.001**
**Group X Posture X Congruency**	**-0.113**	**0.034**	**10.75**	**0.001**
**Instruction X Posture X Congruency**	**0.260**	**0.034**	**58.31**	**<0.001**
Group X Instruction X Movement Context	0.044	0.034	1.64	0.201
Group X Posture X Movement Context	-0.022	0.034	0.42	0.519
Instruction X Posture X Movement Context	-0.051	0.034	2.20	0.138
Group X Congruency X Movement Context	0.023	0.034	0.47	0.493
Instruction X Congruency X Movement Context	0.018	0.034	0.27	0.605
Posture X Congruency X Movement Context	0.004	0.034	0.01	0.915
**Group X Instruction X Posture X Congruency**	**0.129**	**0.034**	**13.83**	**<0.001**
Group X Instruction X Posture X Movement Context	0.054	0.034	2.46	0.117
Group X Instruction X Congruency X Movement Context	-0.010	0.034	0.09	0.766
Group X Posture X Congruency X Movement Context	-0.030	0.034	0.77	0.380
Instruction X Posture X Congruency X Movement Context	-0.043	0.034	1.62	0.203
Group X Instruction X Posture X Congruency X Movement Context	0.000	0.034	0.00	0.993

Summary of the fixed effects in the GLMM of the combined analysis of sighted and blind groups. Coefficients are reported in logit units. Bolded values indicate significance at p < 0.05. Values shown in italics indicate a trend towards significance at p < 0.1. Test statistics are χ^2^ -distributed with 1 degree of freedom.

The full GLMM (see Table 1) revealed a four-way interaction of Group, Instruction, Posture, and Congruency (χ^2^(1) = 13.83, p < 0.001). We followed up on this four-way interaction by first analyzing accuracy separately for each group. In a second step, we then compared congruency effects and performance between the two participant groups by means of post-hoc comparisons of the factor Group for the relevant combinations of Instruction, Posture, and Congruency.

### 2.1. Sighted group: Effects of task instruction

The GLMM on accuracy of the sighted group (Table 2, top) revealed a three-way interaction between Instruction, Posture, and Congruency (χ^2^(1) = 83.53, p < 0.001), suggesting that congruency effects differed in dependence of Instruction and Posture combinations. Indeed, post-hoc comparisons revealed a significant two-way interaction between Posture and Congruency under external (z = 14.77, p < 0.001), but not under anatomical instructions (z = 1.46, p = 0.134). Under external instructions, participants responded more accurately following (anatomically and externally) congruent than incongruent stimulation when the hands were oriented in the same orientation (Fig 2, 2^nd^ column, black circles z = 10.23, p < 0.001). This anatomically coded congruency effect was reversed when the hands were held in different orientations, with more accurate performance for externally congruent (i.e., anatomically incongruent) than externally incongruent (i.e., anatomically congruent) stimulus pairs (Fig 2, 2^nd^ column, gray triangles; z = -7.28, p < 0.001). Under anatomical instructions, an anatomical congruency effect emerged across postures (Fig 2, 1^st^ column; z = -10.10, p < 0.001). In sum, the direction of the tactile congruency effect depended on the instructions: congruency effects were consistent with the instructed spatial coding–anatomical or external–in sighted participants.

**Table 2:** Results of separate GLMMs for each participant group.

Predictor	Estimate	SE	*χ*^2^	*p*
*Sighted group*				
(Intercept)	3.036	0.287		
Instruction	0.030	0.105	0.08	0.773
*Posture*	*-0*.*134*	*0*.*065*	*3*.*17*	*0*.*075*
**Congruency**	**0.509**	**0.067**	**24.82**	***<* .001**
Instruction X Posture	-0.007	0.043	0.02	0.878
**Instruction X Congruency**	**0.301**	**0.043**	**48.57**	***<* .001**
**Posture X Congruency**	**-0.478**	**0.042**	**136.73**	***<* .001**
**Instruction X Posture X Congruency**	**0.390**	**0.042**	**83.53**	***<* .001**
*Blind group*				
(Intercept)	3.551	0.176		
Instruction	-0.036	0.116	0.09	0.759
**Posture**	**-0.271**	**0.068**	**12.74**	**< .001**
**Congruency**	**0.180**	**0.067**	**5.86**	**0.015**
Instruction X Posture	0.042	0.056	0.54	0.461
Instruction X Congruency	0.059	0.056	1.10	0.294
**Posture X Congruency**	**-0.251**	**0.053**	**22.80**	***<* .001**
**Instruction X Posture X Congruency**	**0.127**	**0.052**	**5.82**	**0.016**

Results of separate GLMMs for each participant group with accuracy as dependent variable and fixed effect factors Instruction, Posture, and Congruency. Summary of the fixed effects in the GLMM of the sighted (top) and blind group (bottom). Coefficients are reported in logit units. Bolded values indicate significance at p < 0.05. Values set in italics indicate a trend towards significance at p < 0.1. Test statistics are χ^2^ -distributed with 1 degree of freedom.

### 2.2. Congenitally blind group: Effects of task instruction

The GLMM on blind participants' response accuracy (Table 2, bottom) revealed a significant three-way interaction between Instruction, Posture, and Congruency (χ^2^(1) = 5.82, p = 0.016), suggesting that task instructions modulated the congruency effect also in this participant group. Post-hoc comparisons revealed a two-way interaction between Posture and Congruency under external instructions (z = 4.92, p < 0.001) and a trend towards a two-way interaction under anatomical instructions (z = 1.72, p = 0.085). Under external instructions, participants responded more accurately following (anatomically and externally) congruent than incongruent stimulation when hands were oriented in the same orientation (Fig 2, 4^th^ column, black circles z = 3.75, p = 0.001; corrected for alpha error inflation according to Holm (1979), see Table C in S1 File for uncorrected p-values). This anatomically coded congruency effect was reversed when the hands were held in different orientations, with more accurate performance for externally congruent (i.e., anatomically incongruent) than externally incongruent (i.e., anatomically congruent) stimulus pairs (Fig 2, 4^th^ column, gray triangles; z = -2.55, p = 0.011; Holm corrected). Under anatomical instructions, an anatomical congruency effect emerged across postures (Fig 2, 3^rd^ column; z = 2.83, p = 0.005), and a main effect of Posture, reflecting more accurate responses when the hands were oriented in the same way, emerged across congruency conditions (z = 2.69, p = 0.007). In sum, both task instruction and hand posture modulated congruency effects on accuracy in congenitally blind participants, similarly as in sighted participants.

### 2.3. Comparison of the congruency effect between sighted and congenitally blind participants

The separate analyses of the two groups both revealed an effect of task instructions on the use of tactile reference frames for sighted and blind individuals, Yet, visual inspection of Fig 2 suggests that congruency effects were overall larger in the sighted than in the blind group; this result pattern was evident in reaction times as well (Figs A, B in S1 File). To confirm this interaction effect statistically, we followed up on the significant four-way interaction between Group, Instruction, Posture, and Congruency (see Table 1) with post-hoc comparisons across groups for each combination of Instruction and Posture. Indeed, this analysis revealed significant two-way interactions between Group and Congruency, with larger congruency effects in the sighted than in the blind group for all combinations of Instruction and Posture (Table 3, bold font). Following up on these two-way interactions, we found that blind participants responded more accurately than sighted participants following stimulus pairs that were anatomically incongruent under anatomical instructions and externally incongruent under external instructions (all p < 0.05, bold font in Table 4; as one exception, this difference was only marginally significant after Holm correction for external instructions with differentially oriented hands, corrected p = 0.082). In sum, congenitally blind participants exhibited smaller congruency effects than sighted participants due to better performance in incongruent conditions.

**Table 3:** Results of a second post-hoc analysis of the full GLMM, interaction between Group and Congruency for each combination of Instruction and Posture.

anatomical instruction		external instruction	
same orientation	different orientation	same orientation	different orientation
**z = 3.65, *p* = 0.001**	**z = 4.81, *p* < 0.001**	**z = 2.99, *p* = 0.003**	**z = -3.88, *p* < 0.001**

Results of the post-hoc analysis of the full GLMM giving a direct comparison of performance of sighted and congenitally blind participant groups. p-values are Holm corrected; see Table D in S1 File, for uncorrected p-values, including non-significant ones. Values with p < 0.05 are set in bold font.

**Table 4:** Results of a second post-hoc analysis of the full GLMM, Comparison between groups for each combination of Instruction, Posture, and Congruency.

	anatomical instruction		external instruction	
	same orientation	diff. orientation	same orientation	diff. orientation
congruent	z = -0.13, *p* = n.s.	z = -0.29, *p* = n.s.	z = 0.73, *p* = n.s.	z = 2.40, *p* = n.s.
incongruent	**z = 2.67, *p* = 0.045**	**z = 2.85, *p* = 0.031**	**z = 3.35, *p* = 0.006**	z = -0.09, *p* = n.s.

Results of the post-hoc analysis of the full GLMM giving a direct comparison of performance of sighted and congenitally blind participant groups. p-values are Holm corrected; see Table D in S1 File, for uncorrected p-values, including non-significant ones. Values with p < 0.05 are set in bold font.

### 2.4. Effects of movement context

**Fig 3:**
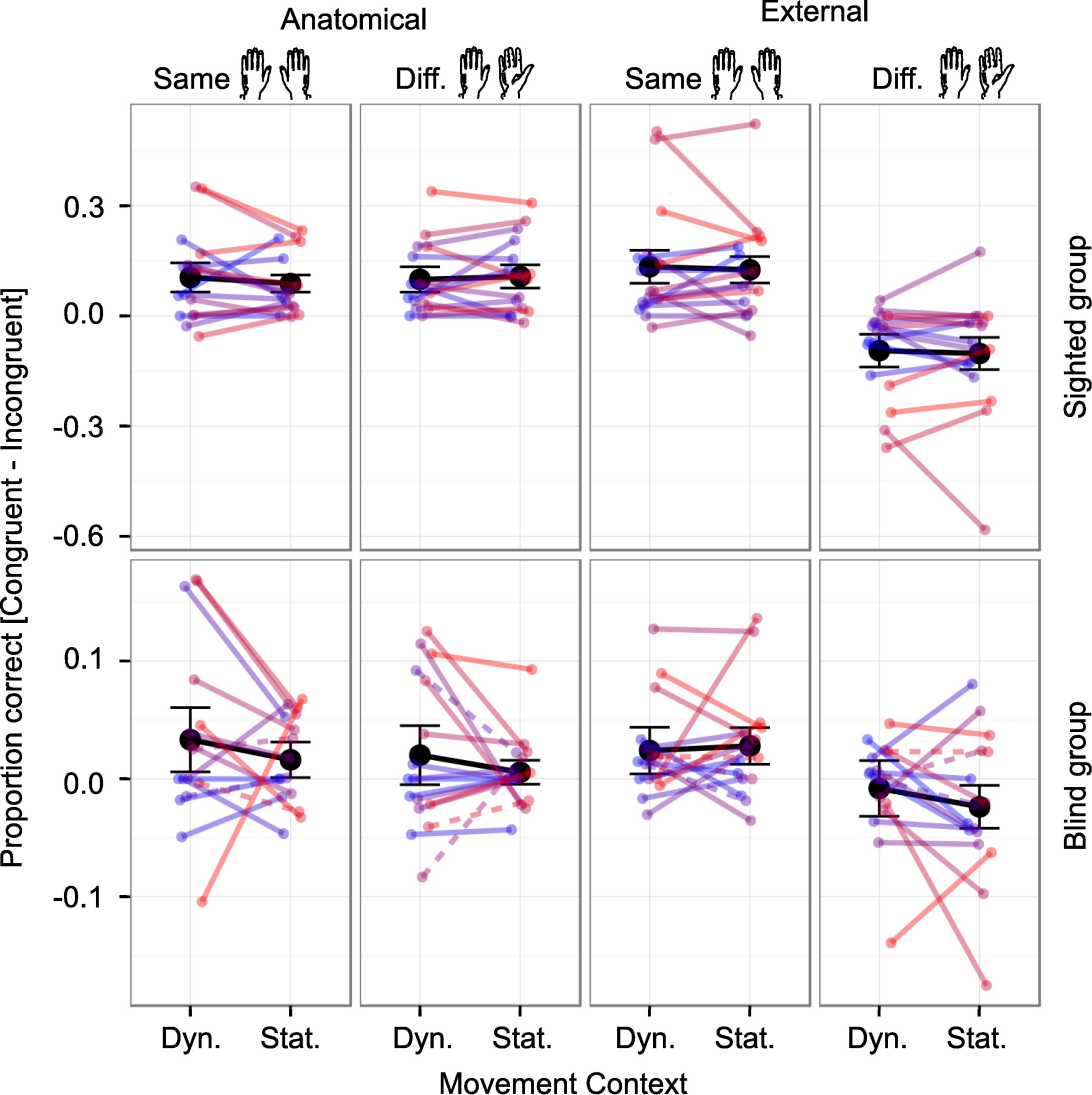
Individual participants’ tactile congruency effects in proportion correct. Response proportions from anatomically incongruent trials were subtracted from response proportions in congruent trials. Congruency effects are plotted for dynamic (“Dyn.”) and static (“Stat.”) contexts with hands held in the same (1^st^ and 3^rd^ column) and in different orientations (2^nd^ and 4^th^ column) under anatomical (1^st^ and 2^nd^ column) and external instructions (3^rd^ and 4^th^ column) in the sighted (top row), and in the congenitally blind group (bottom row). Note that scales differ between groups, reflecting the smaller congruency effects of the blind as compared to the sighted group. Mean congruency effects for each condition are plotted in black, whiskers represent SEM. Each color represents one participant.

Contrary to our expectations, the overall GLMM across both participant groups (see Table 1) did not reveal a significant interaction between Movement Context, Posture, and Congruency or a corresponding higher order interaction (Table 1; all p > 0.05). We observed only a significant main effect of Movement Context (χ^2^(1) = 4.54, p = 0.033) and a trend towards significance for the interaction between Movement Context and Group (χ^2^(1) = 3.47, p = 0.063). To demonstrate that these null effects were not due simply to high variance or a few outliers, Fig 3 shows individual participants' performance.

For completeness, we report a detailed analysis of the influence of Movement Context on reaction times in the supplementary information. This analysis revealed mainly a slowing of reaction times in dynamic contexts; however, none of the emerging interactions lent any support to an influence of Movement Context on the use of tactile reference frames. In sum, we did not find substantial support for our hypothesis that a dynamic context modulates spatial integration in tactile congruency coding of sighted and congenitally blind humans.

## 3. Discussion

The present study investigated whether congenitally blind individuals integrate anatomical and external spatial information during tactile localization in a flexible manner, similar to sighted individuals. To this end, participants localized tactile target stimuli at one hand in the presence of spatially congruent or incongruent distractor stimuli at the other hand. By manipulating hand posture, we varied the congruency of target and distractor locations relative to anatomical and external spatial reference frames. The study comprised two contextual manipulations, both of which influence tactile localization performance in sighted humans according to previous reports. First, we manipulated task context by formulating task instructions with reference to anatomical versus external spatial terms (hand surfaces vs. elevation in space). Second, we manipulated movement context by maintaining participants' hand posture for entire experimental blocks, or alternatively changing hand posture in a trial-by-trial fashion. Task instructions determined the direction of the congruency effect in both groups: Under anatomical instructions, both sighted and congenitally blind participants responded more accurately to anatomically congruent than incongruent target-distractor pairs, whereas under external instructions, participants responded more accurately to externally congruent than to externally incongruent target-distractor pairs. Even though blind participants exhibited congruency effects, interference from incongruent distractors was small compared to the sighted group due to superior performance in incongruent conditions. Movement context, that is, static hand posture versus frequent posture change, did not significantly modulate congruency effects in either experimental group.

### 3.1. Flexible weighting of reference frames according to task instructions in both sighted and blind individuals

Tactile externally coded spatial information has long been presumed to be automatically integrated only by normally sighted and late blind, but not by congenitally blind individuals, [28–30]. In the present study, blind participants’ performance should have been independent of posture and instructions if they had relied on anatomical information alone. Contrary to this assumption, blind participants’ congruency effects changed with posture when the task had been instructed externally, clearly reflecting the use of externally coded information.

The flexible and strategic weighting of anatomical and external tactile information, observed here in both sighted and blind individuals, presumably reflects top-down regulation of spatial integration [16]. In line with this proposal, anatomical and external spatial information are presumed to be available concurrently, as evident, for instance, in event-related potentials [51] and in oscillatory brain activity [52–54]. Furthermore, performance under reference frame conflict, for instance due to hand crossing, is modulated by a secondary task, and this modulation reflects stronger weighting of external information when the secondary task accentuates an external as compared to an anatomical spatial code [8]. The present results, too, demonstrate directed, top-down mediated modulations of spatial weighting, with anatomical task instructions biasing weighting towards anatomical coding, and external instructions biasing weighting towards external coding.

The present study's results seemingly contrast with findings from previous studies. In several previous experiments, hand posture modulated performance of sighted, but not of congenitally blind participants. These results have been interpreted as indicating that sighted, but not congenitally blind individuals integrate external spatial information for tactile localization by default [28–30]. In our experiment, both groups used external coding under external instructions and anatomical coding under anatomical instructions, indicating that developmental vision is not a prerequisite for the ability to flexibly weight information coded in the different reference frames. Thus, sighted and blind individuals do not seem to differ in their general ability to weight information from anatomical and external reference frames. Instead, developmental vision seems to bias the default weighting of anatomical and external information towards the external reference frame, rather than changing the underlying ability to adapt these weights.

### 3.2. Reduced distractor interference in congenitally blind individuals

Congenitally blind participants performed more accurately compared to sighted participants in the presence of incongruent distractors, both when target and distractor were anatomically incongruent under anatomical instructions, and when they were externally incongruent under external instructions. In addition, we had to exclude many sighted participants from the study because they performed at chance level when target and distractor were incongruent relative to the current instruction. Critically, this difficulty of sighted compared to blind participants with the task cannot be due to an inability to discriminate between target and distractor stimuli: neither did discrimination performance significantly differ between sighted and blind groups, nor did we observe a significant correlation between discrimination performance during practice and individual congruency effects. Rather, our results suggest that blind participants were better able to discount spatial information from tactile distractors than sighted individuals, and, accordingly, take into account only the task-relevant spatial information of the target stimulus. Improved selective attention in early blind individuals has previously been reported in auditory, tactile, and auditory-tactile tasks [55–57]. In the tactile domain, this performance difference has been associated with modulations of early ERP components evident in early blind but not in sighted individuals [56,58]. These previous studies employed deviant detection paradigms, in which participants were presented with a rapid stream of stimuli; they reacted towards stimuli matching a specified criterion while withholding responses to non-matching stimuli. As the response criterion usually comprised stimulus location, improved performance in these tasks has often been discussed referring to altered spatial perception in blind individuals [56]. In the present study, the advantage of congenitally blind participants relates to improved selection of a target based on a non-spatial feature; given that targets could occur at any of four locations across the workspace, the improvement of selective attention in congenitally blind individuals was not tied to space. Thus, taken together, congenitally blind individuals appear to have advantages over sighted individuals in both spatial and non-spatial attentional selection in the context of tactile processing.

### 3.3. Comparison of sighted participants’ susceptibility to task instruction with previous tactile localization studies

A previous study tested sighted participants with a similar tactile congruency task as that of the present study and reported that the congruency effect always depended on the external spatial location of tactile stimuli, independent of task instructions when participants responded with the feet [34]. With the same kind of foot responses, the present study demonstrated that the direction of congruency effects is instruction-dependent. This difference may best be explained by the influence of vision on tactile localization in sighted participants: participants had their eyes open in the study of Gallace and colleagues [34], but were blindfolded in the present study. Non-informative vision [59] as well as online visual information about the crossed posture of the own as well as of artificial rubber hands [60–62] seem to evoke an emphasis of the external reference frame. Online visual information about the current hand posture may, thus, have led to the prevalence of the externally coded congruency effect in the study by Gallace and colleagues [34]. In contrast, blindfolding in the present study may have reduced the vision-induced bias towards external space, and, thus, allowed expression of task instruction-induced biases.

### 3.4. Lack of evidence for an effect of movement context on congruency effects

Based on previous findings with other tactile localization paradigms that have manipulated the degree of movement during tactile localization tasks [22–27], we had expected that frequent posture change would emphasize the weighting of an external reference frame in both sighted and blind participants. The reason for the lack of a movement context effect in the present study may be related to our distractor interference paradigm. The need to employ selective attention to suppress task-irrelevant distractor stimuli might have led, as a side effect, to a discounting of task irrelevant factors, such as movement context, in our task. Furthermore, the movements in our task differed from those of previous studies in that they only changed hand orientation, but did not modulate hand position. In contrast, other studies have always required either an arm movement that displaced the hands, or eye and head movements that changed the relative spatial location of touch to the eyes and head.

### 3.5. Summary and conclusion

In sum, we report that both sighted and congenitally blind individuals can flexibly adapt the weighting of anatomical and external information during the encoding of touch, evident in the dependence of tactile congruency effects on task instructions. Our results demonstrate, first, that congenitally blind individuals do not rely on only a single, anatomical reference frame. Instead, they flexibly integrate anatomical and external spatial information. This finding indicates that the use of external spatial information in touch does not ultimately depend on the availability of visual information during development. Second, absence of vision during development appears to improve the ability to select tactile features independent of stimulus location, as demonstrated by reduced distractor interference in congenitally blind participants.

## Supporting information

S1 File Reaction time analysis and other additional analyses. Supporting information contains the results, tables, and figures from the reaction time analysis, from the analysis of data acquired during pre-experimental training, and follow-up analysis on statistical trends.
(PDF)

## References

[1] Alais D, Burr D. The Ventriloquist Effect Results from Near-Optimal Bimodal Integration. Curr Biol. 2004;14: 257–262. doi: 10.1016/j.cub.2004.01.029 14761661 10.1016/j.cub.2004.01.029

[2] Angelaki DE, Gu Y, DeAngelis GC. Multisensory integration: psychophysics, neurophysiology, and computation. Curr Opin Neurobiol. 2009;19: 452–458. doi: 10.1016/j.conb.2009.06.008 19616425 10.1016/j.conb.2009.06.008 PMC2749464

[3] Ernst MO, Banks MS. Humans integrate visual and haptic information in a statistically optimal fashion. Nature. 2002;415: 429–433. doi: 10.1038/415429a 11807554 10.1038/415429a

[4] Pouget A, Deneve S, Duhamel J-R. A computational perspective on the neural basis of multisensory spatial representations. Nat Rev Neurosci. 2002;3: 741–747. doi: 10.1038/nrn914 12209122 10.1038/nrn914

[5] Sober SJ, Sabes PN. Flexible strategies for sensory integration during motor planning. Nat Neurosci. 2005;8: 490–497. doi: 10.1038/nn1427 15793578 10.1038/nn1427 PMC2538489

[6] Trommershäuser J, Körding KP, Landy MS, editors. Sensory Cue Integration. Oxford University Press; 2011.

[7] Badde S, Heed T, Röder B. Integration of anatomical and external response mappings explains crossing effects in tactile localization: A probabilistic modeling approach. Psychon Bull Rev. 2016; 1–18. doi: 10.3758/s13423-015-0918-0 26350763 10.3758/s13423-015-0918-0

[8] Badde S, Röder B, Heed T. Flexibly weighted integration of tactile reference frames. Neuropsychologia. 2015;70: 367–374. doi: 10.1016/j.neuropsychologia.2014.10.001 25447059 10.1016/j.neuropsychologia.2014.10.001

[9] Penfield W, Boldrey E. Somatic motor and sensory representation in the cerebral cortex of man as studied by electrical stimulation. Brain J Neurol. 1937; 389–443.

[10] Clemens IAH, Vrijer MD, Selen LPJ, Gisbergen JAMV, Medendorp WP. Multisensory Processing in Spatial Orientation: An Inverse Probabilistic Approach. J Neurosci. 2011;31: 5365–5377. doi: 10.1523/JNEUROSCI.6472-10.2011 21471371 10.1523/JNEUROSCI.6472-10.2011 PMC6622694

[11] Driver J, Spence C. Cross–modal links in spatial attention. Philos Trans R Soc Lond B Biol Sci. 1998;353: 1319–1331. doi: 10.1098/rstb.1998.0286 9770225 10.1098/rstb.1998.0286 PMC1692335

[12] Heed T, Buchholz VN, Engel AK, Röder B. Tactile remapping: from coordinate transformation to integration in sensorimotor processing. Trends Cogn Sci. 2015;19: 251–258. doi: 10.1016/j.tics.2015.03.001 25843541 10.1016/j.tics.2015.03.001

[13] Holmes NP, Spence C. The body schema and multisensory representation(s) of peripersonal space. Cogn Process. 2004;5: 94–105. doi: 10.1007/s10339-004-0013-3 16467906 10.1007/s10339-004-0013-3 PMC1350799

[14] Maravita A, Spence C, Driver J. Multisensory integration and the body schema: close to hand and within reach. Curr Biol. 2003;13: R531–R539. doi: 10.1016/S0960-9822(03)00449-4 12842033 10.1016/s0960-9822(03)00449-4

[15] Heed T, Backhaus J, Röder B, Badde S. Disentangling the External Reference Frames Relevant to Tactile Localization. PLOS ONE. 2016;11: e0158829 doi: 10.1371/journal.pone.0158829 27391805 10.1371/journal.pone.0158829 PMC4938545

[16] Badde S, Heed T. Towards explaining spatial touch perception: Weighted integration of multiple location codes. Cogn Neuropsychol. 2016;33: 26–47. doi: 10.1080/02643294.2016.1168791 27327353 10.1080/02643294.2016.1168791 PMC4975087

[17] Azañón E, Camacho K, Soto-Faraco S. Tactile remapping beyond space. Eur J Neurosci. 2010;31: 1858–1867. doi: 10.1111/j.1460-9568.2010.07233.x 20584190 10.1111/j.1460-9568.2010.07233.x

[18] Shore DI, Spry E, Spence C. Confusing the mind by crossing the hands. Multisensory Proc. 2002;14: 153–163. doi: 10.1016/S0926-6410(02)00070-8 10.1016/s0926-6410(02)00070-8 12063139

[19] Yamamoto S, Kitazawa S. Reversal of subjective temporal order due to arm crossing. Nat Neurosci. 2001;4: 759–765. doi: 10.1038/89559 11426234 10.1038/89559

[20] Gherri E, Forster B. The orienting of attention during eye and hand movements: ERP evidence for similar frame of reference but different spatially specific modulations of tactile processing. Biol Psychol. 2012;91: 172–184. doi: 10.1016/j.biopsycho.2012.06.007 22750676 10.1016/j.biopsycho.2012.06.007

[21] Gherri E, Forster B. Crossing the hands disrupts tactile spatial attention but not motor attention: evidence from event-related potentials. Neuropsychologia. 2012;50: 2303–2316. doi: 10.1016/j.neuropsychologia.2012.05.034 22683449 10.1016/j.neuropsychologia.2012.05.034

[22] Heed T, Möller J, Röder B. Movement Induces the Use of External Spatial Coordinates for Tactile Localization in Congenitally Blind Humans. Multisensory Res. 2015;28: 173–194. doi: 10.1163/22134808-00002485 10.1163/22134808-00002485 26152057

[23] Hermosillo R, Ritterband-Rosenbaum A, van Donkelaar P. Predicting future sensorimotor states influences current temporal decision making. J Neurosci. 2011;31: 10019–10022. doi: 10.1523/JNEUROSCI.0037-11.2011 21734293 10.1523/JNEUROSCI.0037-11.2011 PMC6703331

[24] Mueller S, Fiehler K. Gaze-dependent spatial updating of tactile targets in a localization task. Front Psychol. 2014;5 doi: 10.3389/fpsyg.2014.00066 24575060 10.3389/fpsyg.2014.00066 PMC3918658

[25] Mueller S, Fiehler K. Effector movement triggers gaze-dependent spatial coding of tactile and proprioceptive-tactile reach targets. Neuropsychologia. 2014;62: 184–193. doi: 10.1016/j.neuropsychologia.2014.07.025 25084225 10.1016/j.neuropsychologia.2014.07.025

[26] Pritchett LM, Carnevale MJ, Harris LR. Reference frames for coding touch location depend on the task. Exp Brain Res. 2012;222: 437–445. doi: 10.1007/s00221-012-3231-4 22941315 10.1007/s00221-012-3231-4

[27] Azañón E, Stenner M-P, Cardini F, Haggard P. Dynamic Tuning of Tactile Localization to Body Posture. Curr Biol. 2015;25: 512–517. doi: 10.1016/j.cub.2014.12.038 25660544 10.1016/j.cub.2014.12.038

[28] Collignon O, Charbonneau G, Lassonde M, Lepore F. Early visual deprivation alters multisensory processing in peripersonal space. Neuropsychologia. 2009;47: 3236–3243. doi: 10.1016/j.neuropsychologia.2009.07.025 19666035 10.1016/j.neuropsychologia.2009.07.025

[29] Röder B, Rösler F, Spence C. Early Vision Impairs Tactile Perception in the Blind. Curr Biol. 2004;14: 121–124. doi: 10.1016/j.cub.2003.12.054 14738733

[30] Röder B, Föcker J, Hötting K, Spence C. Spatial coordinate systems for tactile spatial attention depend on developmental vision: evidence from event-related potentials in sighted and congenitally blind adult humans. Eur J Neurosci. 2008;28: 475–483. doi: 10.1111/j.1460-9568.2008.06352.x 18702719 10.1111/j.1460-9568.2008.06352.x

[31] Heed T, Röder B. Motor coordination uses external spatial coordinates independent of developmental vision. Cognition. 2014;132: 1–15. doi: 10.1016/j.cognition.2014.03.005 24727423 10.1016/j.cognition.2014.03.005

[32] Crollen V, Albouy G, Lepore F, Collignon O. How visual experience impacts the internal and external spatial mapping of sensorimotor functions. Sci Rep. 2017;7: 1022 doi: 10.1038/s41598-017-01158-9 28432316 10.1038/s41598-017-01158-9 PMC5430802

[33] Bottini R, Crepaldi D, Casasanto D, Crollen V, Collignon O. Space and time in the sighted and blind. Cognition. 2015;141: 67–72. doi: 10.1016/j.cognition.2015.04.004 25935747 10.1016/j.cognition.2015.04.004

[34] Gallace A, Soto-Faraco S, Dalton P, Kreukniet B, Spence C. Response requirements modulate tactile spatial congruency effects. Exp Brain Res. 2008;191: 171–186. doi: 10.1007/s00221-008-1510-x 18709500 10.1007/s00221-008-1510-x

[35] Soto-Faraco S, Ronald A, Spence C. Tactile selective attention and body posture: Assessing the multisensory contributions of vision and proprioception. Percept Psychophys. 2004;66: 1077–1094. doi: 10.3758/BF03196837 15751467 10.3758/bf03196837

[36] Millar S. Self-Referent and Movement Cues in Coding Spatial Location by Blind and Sighted Children. Perception. 1981;10: 255–264. doi: 10.1068/p100255 7329748 10.1068/p100255

[37] Eardley AF, van Velzen J. Event-related potential evidence for the use of external coordinates in the preparation of tactile attention by the early blind. Eur J Neurosci. 2011;33: 1897–1907. doi: 10.1111/j.1460-9568.2011.07672.x 21545656 10.1111/j.1460-9568.2011.07672.x

[38] Schicke T, Bauer F, Röder B. Interactions of different body parts in peripersonal space: how vision of the foot influences tactile perception at the hand. Exp Brain Res. 2009;192: 703–715. doi: 10.1007/s00221-008-1587-2 18841353 10.1007/s00221-008-1587-2

[39] Bates D, Maechler M, Bolker B, Walker S. lme4: Linear mixed-effects models using Eigen and S4 [Internet]. 2014. Available: http://CRAN.R-project.org/package=lme4

[40] Singmann H, Bolker B, Westfall J. afex: Analysis of Factorial Experiments [Internet]. 2015. Available: http://CRAN.R-project.org/package=afex

[41] Lenth RV, Hervé M. lsmeans: Least-Squares Means [Internet]. 2015. Available: http://CRAN.R-project.org/package=lsmeans

[42] Wickham H, Francois R, Henry L, Müller K. dplyr: A Grammar of Data Manipulation [Internet]. 2017. Available: https://CRAN.R-project.org/package=dplyr

[43] Wickham H. ggplot2: elegant graphics for data analysis [Internet]. Springer New York; 2009 Available: http://had.co.nz/ggplot2/book

[44] Jaeger TF. Categorical Data Analysis: Away from ANOVAs (transformation or not) and towards Logit Mixed Models. J Mem Lang. 2008;59: 434–446. doi: 10.1016/j.jml.2007.11.007 19884961 10.1016/j.jml.2007.11.007 PMC2613284

[45] Bolker BM, Brooks ME, Clark CJ, Geange SW, Poulsen JR, Stevens MHH, et al Generalized linear mixed models: a practical guide for ecology and evolution. Trends Ecol Evol. 2009;24: 127–135. doi: 10.1016/j.tree.2008.10.008 19185386 10.1016/j.tree.2008.10.008

[46] Barr DJ, Levy R, Scheepers C, Tily HJ. Random effects structure for confirmatory hypothesis testing: Keep it maximal. J Mem Lang. 2013;68: 255–278. doi: 10.1016/j.jml.2012.11.001 24403724 10.1016/j.jml.2012.11.001 PMC3881361

[47] Bates D, Kliegl R, Vasishth S, Baayen H. Parsimonious Mixed Models. ArXiv150604967 Stat. 2015; Available: http://arxiv.org/abs/1506.04967

[48] Brandes J, Heed T. Reach Trajectories Characterize Tactile Localization for Sensorimotor Decision Making. J Neurosci. 2015;35: 13648–13658. doi: 10.1523/JNEUROSCI.1873-14.2015 26446218 10.1523/JNEUROSCI.1873-14.2015 PMC6605379

[49] Pinheiro JC, Bates D. Mixed-Effects Models in S and S-PLUS. New York, NY: Springer; 2000.

[50] Holm S. A Simple Sequentially Rejective Multiple Test Procedure. Scand J Stat. 1979;6: 65–70.

[51] Heed T, Röder B. Common anatomical and external coding for hands and feet in tactile attention: evidence from event-related potentials. J Cogn Neurosci. 2010;22: 184–202. doi: 10.1162/jocn.2008.21168 19199399 10.1162/jocn.2008.21168

[52] Buchholz VN, Jensen O, Medendorp WP. Parietal oscillations code nonvisual reach targets relative to gaze and body. J Neurosci. 2013;33: 3492–3499. doi: 10.1523/JNEUROSCI.3208-12.2013 23426676 10.1523/JNEUROSCI.3208-12.2013 PMC6619549

[53] Buchholz VN, Jensen O, Medendorp WP. Multiple reference frames in cortical oscillatory activity during tactile remapping for saccades. J Neurosci. 2011;31: 16864–16871. doi: 10.1523/JNEUROSCI.3404-11.2011 22090512 10.1523/JNEUROSCI.3404-11.2011 PMC6633300

[54] Schubert JTW, Buchholz VN, Föcker J, Engel AK, Röder B, Heed T. Oscillatory activity reflects differential use of spatial reference frames by sighted and blind individuals in tactile attention. NeuroImage. 2015;117: 417–428. doi: 10.1016/j.neuroimage.2015.05.068 26032885 10.1016/j.neuroimage.2015.05.068

[55] Collignon O, Renier L, Bruyer R, Tranduy D, Veraart C. Improved selective and divided spatial attention in early blind subjects. Brain Res. 2006;1075: 175–182. doi: 10.1016/j.brainres.2005.12.079 16460716 10.1016/j.brainres.2005.12.079

[56] Hötting K, Rösler F, Röder B. Altered auditory-tactile interactions in congenitally blind humans: an event-related potential study. Exp Brain Res. 2004;159: 370–381. doi: 10.1007/s00221-004-1965-3 15241575 10.1007/s00221-004-1965-3

[57] Röder B, Teder-Salejarvi W, Sterr A, Rösler F, Hillyard SA, Neville HJ. Improved auditory spatial tuning in blind humans. Nature. 1999;400: 162–166. doi: 10.1038/22106 10408442 10.1038/22106

[58] Forster B, Eardley AF, Eimer M. Altered tactile spatial attention in the early blind. Brain Res. 2007;1131: 149–154. doi: 10.1016/j.brainres.2006.11.004 17173872 10.1016/j.brainres.2006.11.004

[59] Newport R, Rabb B, Jackson SR. Noninformative Vision Improves Haptic Spatial Perception. Curr Biol. 2002;12: 1661–1664. doi: 10.1016/S0960-9822(02)01178-8 12361568 10.1016/s0960-9822(02)01178-8

[60] Azañón E, Soto-Faraco S. Alleviating the “crossed-hands” deficit by seeing uncrossed rubber hands. Exp Brain Res. 2007;182: 537–548. doi: 10.1007/s00221-007-1011-3 17643239 10.1007/s00221-007-1011-3

[61] Cadieux ML, Shore DI. Response demands and blindfolding in the crossed-hands deficit: an exploration of reference frame conflict. Multisensory Res. 2013;26: 465–482. doi: 10.1163/22134808-00002423 10.1163/22134808-00002423 24649530

[62] Pavani F, Spence C, Driver J. Visual Capture of Touch: Out-of-the-Body Experiences With Rubber Gloves. Psychol Sci. 2000;11: 353–359. doi: 10.1111/1467-9280.00270 11228904 10.1111/1467-9280.00270

