# Supplementary material for: Task demands affect spatial reference frame weighting during tactile localization in sighted and congenitally blind adults
Source: PLoS One. 2017 Dec 11;12(12):e0189067. doi: 10.1371/journal.pone.0189067 (PMC5724835; doi:10.1371/journal.pone.0189067)
Supplement: S1 File — Supporting information contains the results, tables, and figures from the reaction time analysis, from the analysis of data acquired during pre-experimental training, and follow-up analysis on statistical trends. (PDF) [file pone.0189067.s001.pdf]

Supporting information

to accompany **Task demands affect spatial reference frame weighting during tactile localization in sighted and congenitally blind adults**

Jonathan T.W. Schubert, Stephanie Badde Brigitte Röder, and Tobias Heed

Reaction time analysis

Methods A:

We analyzed reaction times (RT) for trials with correct responses using linear mixed models (LMM). To prevent that the fitted model's residuals violated normality and homoscedasticity assumptions, reaction times were box-cox transformed (Box & Cox, 1964) with lambda = -0.06. For random effects over participants, we used the maximal combination of factors that allowed convergence for all fitted mixed models of the experiment (Barr, Levy, Scheepers, & Tily, 2013; Bates, Kliegl, Vasishth, & Baayen, 2015). Reliable convergence was achieved when we included random intercepts and slopes per participants for each main effect, but not for interactions. Significance of fixed effects was assessed with likelihood ratio tests comparing the model with the maximal fixed effects structure and a reduced model without the fixed effect of interest (Pinheiro & Bates, 2000). These comparisons were calculated using the afex package (Singmann, Bolker, & Westfall, 2015), and employed Type III sums of squares and sum-to-zero contrasts. Fixed effects were considered significant at $p < 0.05$. Post-hoc comparisons of significant interactions were conducted using approximate z-tests on the estimated least square means (LSM, lsmeans package; Lenth & Hervé, 2015). The resulting p-values were corrected for multiple comparisons following the procedure proposed by Holm (1979). To assess whether the overall result pattern differed between groups, we fitted a GLMM with the fixed between-subject factor Group (sighted, blind) and fixed within-subjects factors Instruction (anatomic, external), Posture (same, different), Congruency (congruent, incongruent), and Movement Context (static, dynamic). Congruency was defined relative to anatomical locations for statistical analysis and figures. Subsequently, to reduce LMM complexity and to ease interpretability, we conducted separate analyses for each participant group including the same within-subject fixed effects as before.

Results A: Task instruction (sighted group)

The result pattern of sighted participants' reaction times was qualitatively similar to that of the accuracy results. The LMM (Table A) showed a three-way interaction between Instruction, Posture, and Congruency ($\chi^2(1) = 52.98$, $p < 0.001$), indicating that instructions affected the congruency effect. Post-hoc comparisons yielded a two-way interaction between Posture and Congruency under anatomical ($z = 9.99$, $p < 0.001$) and external instructions ($z = 20.25$, $p < 0.001$).

With both hands in the same orientation (Fig. A, black circles), sighted participants responded faster to (anatomically and externally) congruent stimulus pairs than to incongruent stimulus pairs, regardless of instruction (external instruction: $z = 10.47$, $p < 0.001$; anatomical: $z = 10.37$, $p < 0.001$).

With differently oriented hands (Fig. B, gray triangles), an anatomical congruency effect emerged under anatomical instructions ($z = 2.14$, $p = 0.034$), though this effect was smaller than in the same orientation conditions. In contrast, an externally coded congruency effect emerged under external instructions ($z = -6.23$, $p < 0.001$).

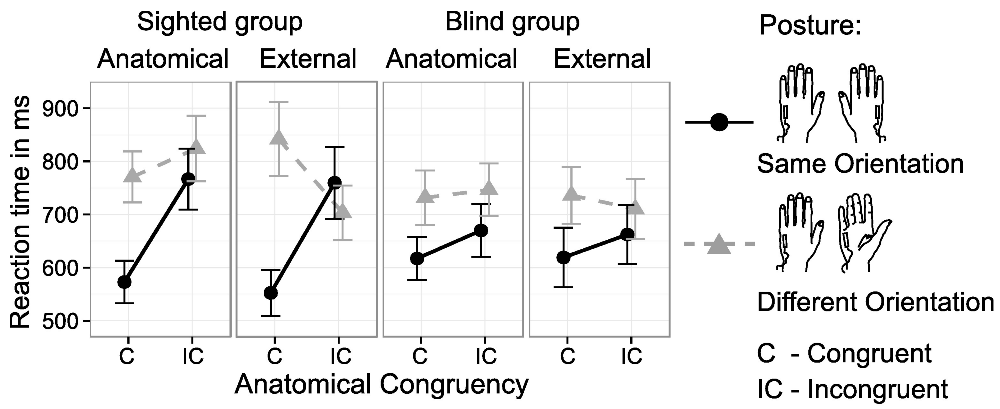

Figure A. Reaction times in the tactile congruency task collapsed over static and dynamic movement conditions. Sighted (2 left columns) and congenitally blind participants (2 right columns) were instructed to localize tactile targets either relative to their anatomical (1st and 3rd column) or relative to their external spatial location (2nd and 4th column). Hands were placed in the same (black circles) and in different orientations (grey triangles). Tactile distractors were presented to anatomically congruent (C) and incongruent (IC) locations of the other hand and had to be ignored. Congruency is defined in anatomical terms (see Fig. 1). Accordingly, with differently oriented hands, anatomically congruent stimulus pairs are incongruent in external space and vice versa. Whiskers represent the standard error of the mean.

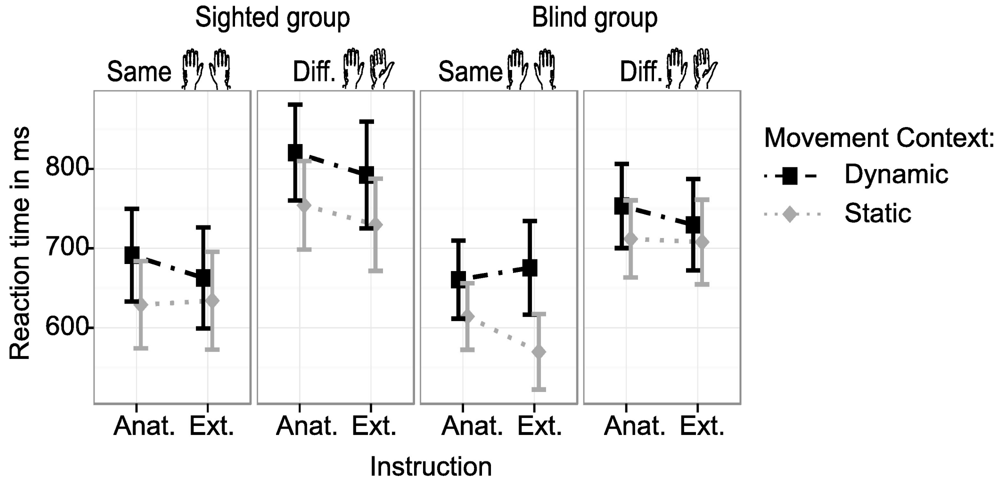

Figure B. **Effect of Movement Context on reaction times, collapsed over congruency conditions.** Participants localized tactile targets with hands in the same (1st and 3rd column) and in different orientations (2nd and 4th column), under anatomical ("Anat.") and under external instructions ("Ext."), in the context of static blockwise posture changes (gray diamonds) and in the context of frequent trial-by-trial posture changes (black squares). Error bars show standard errors of the mean.

Results B: Movement context (sighted group)

For reaction times, the LMM revealed a main effect of Movement Context ($\chi^2(1) = 7.60$, $p = 0.005$), indicating that sighted participants responded overall faster in the static than in the dynamic movement context. In addition, there was a trend for a three-way interaction between Instruction, Posture, and Movement Context ($\chi^2(1) = 3.49$, $p = 0.062$), due to a larger reaction time gain under anatomical than under external instructions, but only when the hands were in the same orientation: in this latter case, the two-way interaction of Instruction and Movement Context was significant ($z = 2.51$, $p = 0.024$); participants responded faster in the static than in the dynamic condition under anatomical instructions ($z = 3.72$, $p = 0.008$), and this effect was reduced under external instructions ($z = 2.12$, $p = 0.034$). With the hands in different orientations, the two-way interaction between Instruction and Movement was not significant ($z = -0.14$, $p = 0.886$). Thus, an effect of Movement Context was evident in all conditions, and the marginal three-way interaction of Instruction, Posture, and Movement Context suggested that it was mainly due to the instruction effects for the same orientation condition. In sum, although frequent movement generally slowed reaction times, movement context did not significantly affect the congruency effect in either of the present study's dependent measures, accuracy or reaction times.

Results C: Task instruction (congenitally blind group)

Reaction time analysis corroborated accuracy results. The LMM on blind participants' reaction times revealed a three-way interaction between Instruction, Posture, and Congruency ($\chi^2(1) = 7.26$, $p = 0.007$; see Fig. A, 3rd and 4th column). Post-hoc comparisons revealed a two-way interaction between Posture and Congruency under anatomical ($z = 2.79$, $p = 0.005$) and external instructions ($z = 6.65$, $p < 0.001$).

With hands held in the same orientation, blind participants responded significantly faster to (anatomically and externally) congruent than incongruent stimulus pairs under anatomical ($z = 4.41$, $p < 0.001$) and under external instructions ($z = 4.26$, $p < 0.001$).

With differently oriented hands, no significant congruency effect was observed under anatomical instructions ($z = 1.55$, $p = 0.120$). The congruency effect was reversed under external instructions relative to the congruency effect when the hands were held in the same orientation, with faster responses to externally congruent (but anatomically incongruent) than externally incongruent (but anatomically congruent) stimulus pairs ($z = -2.48$, $p = 0.026$).

In sum, both accuracy and reaction times of blind participants reflected an influence of task instructions on tactile-spatial congruency coding.

Results D: Movement context (congenitally blind group)

Reaction times

The LMM on reaction times revealed a three-way interaction between Instruction, Posture, and Movement Context ($\chi^2(1) = 22.23$, $p < 0.001$), suggesting a modulation of the congruency effect on reaction times by Movement Context. Following up on the three-way interaction, post-hoc

comparisons yielded a two-way interaction between Instruction and Movement Context with the hands in the same ($z = -5.30$, $p < 0.001$), but not in different orientations ($z = 1.39$, $p = 0.163$).

Blind participants responded significantly faster in the static than in the dynamic condition with hands in the same orientation under external instructions ($z = 5.01$, $p < 0.001$), but only marginally faster under anatomical instructions ($z = 2.27$, $p = 0.069$). No significant difference between conditions was observed with differently oriented hands under either instruction (anatomical: $z = 1.58$, $p = 0.113$; external: $z = 0.85$, $p = 0.391$).

An influence of frequent posture changes on the weighting of external spatial information should be evident in a modulation of congruency effects with the hands oriented differently rather than in the same posture. Just like for accuracy, the corresponding four-way interaction of Instruction, Posture, Congruency, and Movement Context was not significant for reaction times ($\chi^2(1) = 0.05$, $p = 0.830$). Although there was a two-way interaction of Congruency and Movement Context ($\chi^2(1) = 17.92$, $p < 0.001$) with a congruency effect in the static ($z = 4.02$, $p < 0.001$), but not in the dynamic condition ($z = 0.47$, $p = 0.635$), this interaction does not differentiate between instructions and postures. As had been the case with accuracy, visual inspection of the reaction times result pattern (Fig. C, bottom) suggested that – as hypothesized – posture may have modulated the congruency effect in the expected direction, but a lack of power may have prevented statistical significance. Therefore, we conducted hypothesis-driven post-hoc tests of the relevant conditions (i.e., conditions with differently oriented hands). Under anatomical instructions, a congruency effect was present in the static movement context ($z = 2.06$, $p = 0.047$), but not in the dynamic movement context ($z= 0.13$, $p = 0.900$). Under external instructions, a significant congruency effect was not evident in the static ($z = -0.96$, $p = 0.335$), but in the dynamic context ($z = -3.06$, $p = 0.009$), with faster responses for externally congruent (but anatomically incongruent) than externally incongruent (but anatomically congruent) stimulus pairs. These comparisons imply that an anatomical congruency effect was present under anatomical instructions only in the static context, and an external congruency effect under external instruction only in the dynamic context. While the presence of these partial instruction-related effects are in line with our hypotheses, the lack of a significant higher-order interaction precludes any strong interpretation of these direct statistical tests. Specifically, congruency effects decreased numerically in all instruction and posture conditions (see Fig. C, bottom), an effect that does not comply with our hypothesis about an effect of the movement context. Furthermore, congruency effects were quite variable across blind participants, as evident in individual modulations of congruency effects (see Fig. C), further corroborating the conclusion that reliable modulations of congruency by movement context were not present on the group level.

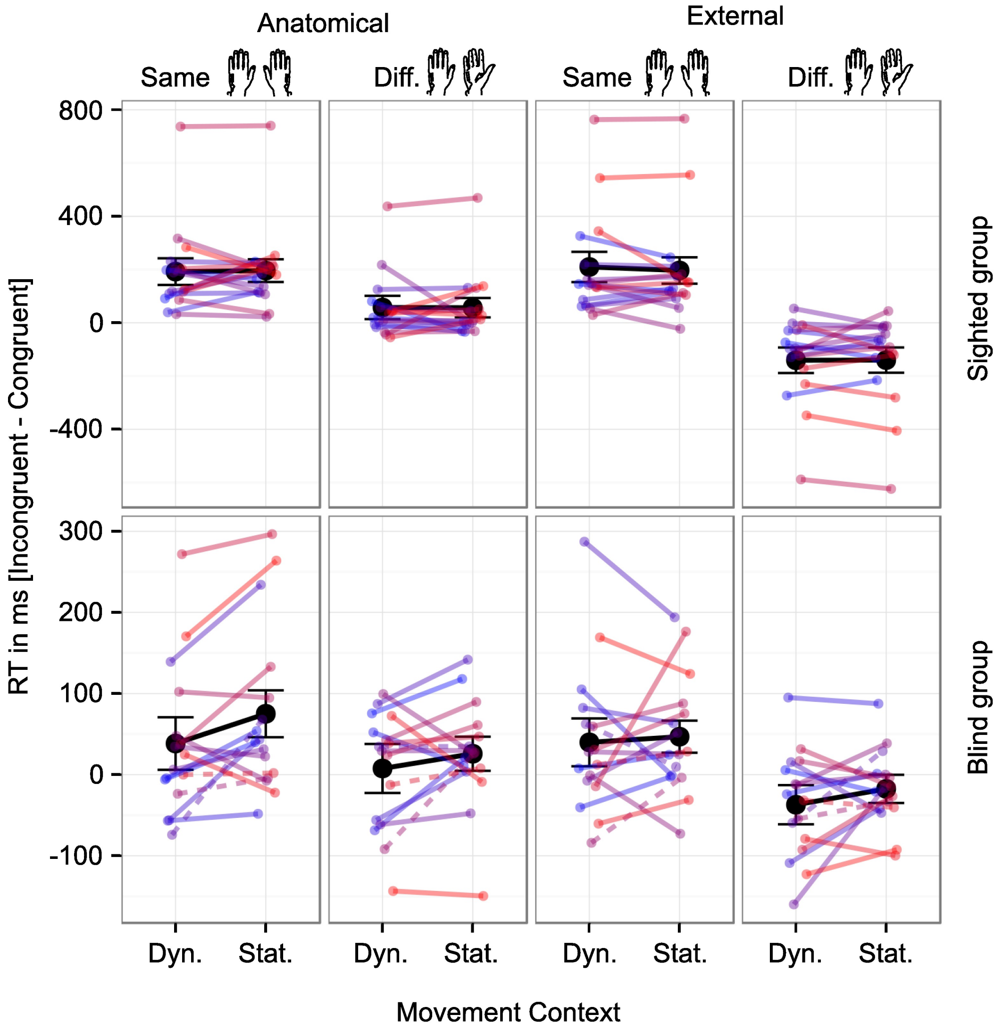

Figure C. Individual participants' tactile congruency effects in reaction times. Responses from anatomically incongruent trials were subtracted from responses in congruent trials. Congruency effects are plotted for dynamic ("Dyn.") and static ("Stat.") contexts with hands in the same (1st and 3rd column) and in different orientations (2nd and 4th column) under anatomical (1st and 2nd column) and external instructions (3rd and 4th column) in the sighted (top row) and in the congenitally blind group (bottom row). Note that scales differ between groups because congruency effects in the blind group were smaller than in the sighted group. Mean congruency effects for each condition are plotted in black, whiskers represent SEM. Each color represents one participant.

Results E: Movement context follow-up (congenitally blind group)

Accuracy: follow-up analysis on trends

The GLMM on blind participants' accuracy showed a main effect of movement context (mentioned in the main text). Besides this main effect, there was a trend for a three-way interaction between Instruction, Posture, and Movement Context ($\chi^2(1) = 3.75$, $p = 0.053$). Following up on this trend, post-hoc comparisons yielded a trend for a two-way interaction between Instruction and Movement Context with hands in different orientations ($z = -2.15$, $p = 0.063$), but not with hands in the same orientation ($z = 0.73$, $p = 0.465$).

Following up on the trend for a two-way interaction between Instruction by Movement Context revealed that static and dynamic conditions did not significantly differ under either instruction when the hands were in the same orientation, (anatomical: $z = 0.50$, $p = 0.618$; external: $z = 1.36$, $p = 0.173$). With differently oriented hands, participants responded more accurately in the static than in the dynamic condition under anatomical ($z = 3.09$, $p = 0.004$), but not under external instructions ($z = 0.37$, $p = 0.713$). Thus, the performance pattern was suggestive of some selective effects of task instructions on accuracy, but the statistical results were only marginal.

We had hypothesized that frequent posture changes would emphasize the weighting of external spatial information. Such an effect would be evident in a modulation of congruency effects emerging with the hands in different postures. The corresponding interaction in the GLMM, the four-way interaction of Instruction, Posture, Congruency, and Movement Context, was not significant ($\chi^2(1) = 0.63$, $p = 0.427$). Yet, visual inspection of Fig. 3 suggested that an effect may be present, but remained non-significant due to lack of power of a GLMM with several factors. Therefore, we performed hypothesis-based post-hoc tests for conditions with differently oriented hands. In the static condition, no significant congruency effect was present under anatomical instructions ($z = 0.52$, $p = 0.605$), but a trend for a congruency effect was observed under external instructions ($z = -2.34$, $p = 0.078$). In the dynamic condition, no significant congruency effect was present under anatomical instructions ($z = 1.15$, $p = 0.252$) and external instructions ($z = -1.48$, $p = 0.138$). Thus, even when directly comparing movement conditions while ignoring other experimental conditions, the hypothesis that a dynamic context modulates spatial integration in tactile congruency coding of congenitally blind humans did not receive any substantial support.

Additional analyses supplementing accuracy analysis of the main text

Results F: Target-distractor discrimination during practice

Before starting the experiment, participants completed two blocks of 24 trials, in which they were asked to discriminate target from distractor stimulus. In each trial, either a target or distractor stimulus was randomly presented to one of the four possible stimulus locations. Participants were asked to lift the toes when they perceived a target and the heels when they perceived a distractor

stimulus. In case participants did not give a correct response, they received auditory feedback. Due to technical reasons data from one sighted and from one blind participant were not recorded during this practice session. Thus, data from 15 sighted and 15 congenitally blind participants were analyzed. Reaction times (of correct trials only) and accuracy were analyzed using a (generalized) linear mixed model with the fixed effect factors Group and Block (see main text for details). The models reliably converged when we included random intercepts and slopes per participant for Block. For accuracy, a GLMM with a binomial link function was used. Reaction times were box-cox transformed with lambda = 0.18 to prevent that the fitted model's residuals violated normality and homoscedasticity assumptions.

Performance is shown in figure D. The (G)LMM revealed that participants responded more accurately and faster in the second (mean accuracy: 94.43 % correct; mean RT: 459 ms) compared to the first (mean accuracy: 97.04 % correct; mean RT: 517 ms) training block (fixed effect Block on accuracy: $\chi2(1) = 4.28$, $p = 0.039$; RT: $\chi2(1) = 12.40$, $p < 0.001$). Critically, the analyses did neither yield significant differences between groups (fixed effect Group on accuracy: $\chi2(1) = 0.73$, $p = 0.392$; RT: $\chi2(1) = 0.08$, $p = 0.778$) nor significant interactions between the fixed effect factors Group and Block (accuracy: $\chi2(1) = 0.74$, $p = 0.389$ RT: $\chi2(1) = 0.06$, $p = 0.801$).

In sum, visual experience did not significantly influence discrimination performance during the pre-experimental training.

Moreover, to test whether discrimination performance related to performance in the tactile congruency task discrimination performance during practice was correlated with individual congruency effects, that is, congruent versus incongruent trial performance. This correlation was calculated separately for each hand position, instruction, and group. However, none of these correlations reached statistical significance (all $p \geq 0.127$; uncorrected; reaction times and accuracies).

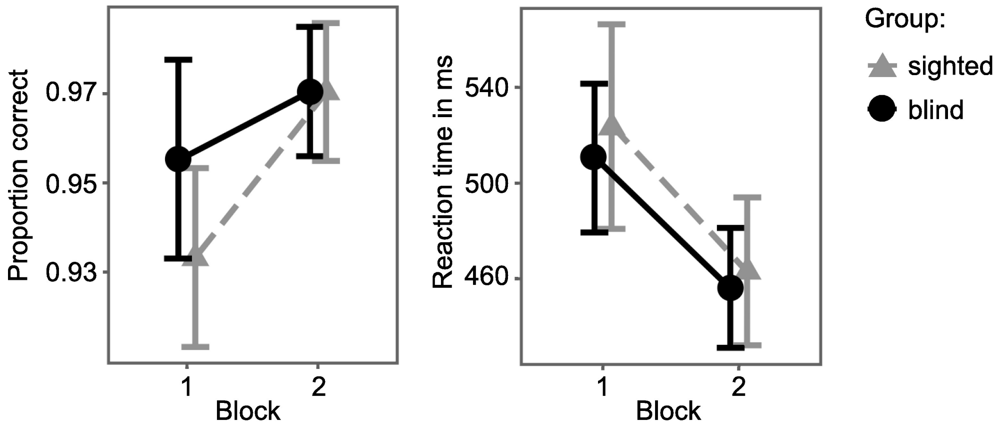

Figure D. **Accuracy (left) and reaction times (right) during pre-experimental training.** Sighted (gray triangles) and congenitally blind (black circles) were asked to discriminate single vibrotactile stimuli randomly presented to one of their hands. Stimuli consisted either of a target or a distractor stimulus pattern (see methods section for details). Whereas participants responded faster and more accurate in the second (right in each panel) compared to the first block (left in each panel), performance did not significantly differ between groups. Whiskers represent SEM.

Table A. Statistical results from reaction time analysis. Summary of the fixed effects in the LMM of the sighted group, of the blind group, and of the combined analysis. Bold values indicate significance at $p < 0.05$. Italic values indicate a trend towards significance at $p < 0.1$. Test statistics are $\chi 2$ -distributed with 1 degree of freedom.

Predictor	Estimate	SE	χ^2	*p*
Both groups				
(Intercept)	5.321	0.031		
Group	0.006	0.031	0.03	0.852
Instruction	0.012	0.01	1.24	0.265
**Posture**	**0.054**	**0.004**	**60.45**	**<.001**
**Congruency**	**-0.022**	**0.004**	**20.76**	**<.001**
**Movement Context**	**0.028**	**0.007**	**12.88**	**<.001**
Group X Instruction	0.005	0.01	0.28	0.594
Group X Posture	0.004	0.004	1.13	0.287
Instruction X Posture	0.001	0.002	0.20	0.658
**Group X Congruency**	**-0.011**	**0.004**	**6.71**	**0.010**
**Instruction X Congruency**	**-0.011**	**0.002**	**53.05**	**<.001**
**Posture X Congruency**	**0.031**	**0.002**	**417.83**	**<.001**
Group X Movement Context	0.001	0.007	0.03	0.868
Instruction X Movement Context	-0.001	0.002	0.30	0.581
**Posture X Movement Context**	**-0.007**	**0.002**	**20.52**	**<.001**
**Congruency X Movement Context**	**0.006**	**0.002**	**14.08**	**<.001**
Group X Instruction X Posture	0.002	0.002	1.95	0.162
**Group X Instruction X Congruency**	**-0.005**	**0.002**	**11.45**	**0.001**
**Group X Posture X Congruency**	**0.018**	**0.002**	**136.92**	**<.001**
**Instruction X Posture X Congruency**	**-0.011**	**0.002**	**52.72**	**<.001**
**Group X Instruction X Movement Context**	**0.005**	**0.002**	**9.47**	**0.002**
**Group X Posture X Movement Context**	**0.006**	**0.002**	**17.73**	**<.001**
Instruction X Posture X Movement Context	*0.003*	*0.002*	*2.93*	*0.087*
Group X Congruency X Movement Context	*-0.003*	*0.002*	*3.49*	*0.062*
Instruction X Congruency X Movement Context	0.001	0.002	0.43	0.513
Posture X Congruency X Movement Context	-0.001	0.002	0.50	0.479
**Group X Instruction X Posture X Congruency**	**-0.006**	**0.002**	**13.67**	**<.001**
**Group X Instruction X Posture X Movement Context**	**-0.007**	**0.002**	**20.51**	**<.001**
Group X Instruction X Congruency X Movement Context	0.000	0.002	0.04	0.849
Group X Posture X Congruency X Movement Context	0.000	0.002	0.01	0.927
Instruction X Posture X Congruency X Movement Context	0.001	0.002	0.25	0.618
Group X Instruction X Posture X Congruency X Movement Context	0.001	0.002	0.59	0.444
Sighted group				
(Intercept)	5.327	0.046		
Instruction	0.017	0.014	1.34	0.247
**Posture**	**0.058**	**0.005**	**33.84**	**<.001**
**Congruency**	**-0.033**	**0.007**	**14.39**	**<.001**
**Movement Context**	**0.029**	**0.009**	**7.60**	**0.006**
Instruction X Posture	0.003	0.002	1.58	0.209
**Instruction X Congruency**	**-0.016**	**0.002**	**50.23**	**<.001**
**Posture X Congruency**	**0.049**	**0.002**	**447.97**	**<.001**
Instruction X Movement Context	*0.004*	*0.002*	*2.77*	*0.096*
Posture X Movement Context	0.000	0.002	0.04	0.834
Congruency X Movement Context	0.003	0.002	1.56	0.212
**Instruction X Posture X Congruency**	**-0.017**	**0.002**	**52.98**	**<.001**
Instruction X Posture X Movement Context	*-0.004*	*0.002*	*3.49*	*0.062*
Instruction X Congruency X Movement Context	0.001	0.002	0.32	0.574
Posture X Congruency X Movement Context	-0.001	0.002	0.28	0.598
Instruction X Posture X Congruency X Movement Context	0.002	0.002	0.74	0.389
Blind group				
(Intercept)	5.316	0.042		
Instruction	0.006	0.015	0.17	0.678
**Posture**	**0.049**	**0.006**	**26.58**	**<.001**
**Congruency**	**-0.011**	**0.004**	**5.17**	**0.023**
**Movement Context**	**0.027**	**0.01**	**5.50**	**0.019**
Instruction X Posture	-0.001	0.002	0.54	0.462
**Instruction X Congruency**	**-0.006**	**0.002**	**8.63**	**0.003**
**Posture X Congruency**	**0.013**	**0.002**	**44.35**	**<.001**
**Instruction X Movement Context**	**-0.005**	**0.002**	**7.47**	**0.006**
**Posture X Movement Context**	**-0.013**	**0.002**	**43.44**	**<.001**
**Congruency X Movement Context**	**0.008**	**0.002**	**17.92**	**<.001**
**Instruction X Posture X Congruency**	**-0.005**	**0.002**	**7.26**	**0.007**
**Instruction X Posture X Movement Context**	**0.009**	**0.002**	**22.23**	**<.001**
Instruction X Congruency X Movement Context	0.001	0.002	0.12	0.724
Posture X Congruency X Movement Context	-0.001	0.002	0.22	0.641
Instruction X Posture X Congruency X Movement Context	0.000	0.002	0.05	0.830

Table. a, Reaction times. Interaction between Group and Congruency for each instruction and posture. Values with $p < 0.05$ are in bold font.

anatomical instruction		external instruction	
same posture	different posture	same posture	different posture
**z = -5.89, *p* < 0.001**	z = -0.82, *p* = 0.412	**z = -6.10, *p* < 0.001**	**z = 3.65, *p* < 0.001**

b, Comparison between groups for each combination of Instruction and Posture for congruent (C) and incongruent trials (IC).

	anatomical instruction		external instruction	
	same posture	different posture	same posture	different posture
C	z = 0.75, *p* = 0.454	z = -0.47, *p* = 0.641	z = 0.86, *p* = 0.385	z = -0.58, *p* = 0.564
IC	z = -1.05, *p* = 0.296	z = -0.71, *p* = 0.475	z = -0.74, *p* = 0.459	z = 0.42, *p* = 0.678

Table C. Corrected and uncorrected p-values for post-hoc comparisons following within group analysis reported in the main text. P-values were adjusted for multiple comparisons following the procedure proposed by Holm (1979). Instead, one could also compare observed p-values with the adjusted α-level. α-adjustments are, thus, indicated in the last column. Note that comparison terms were sorted with p-values in ascending order for each block of comparison terms.

comparison terms	z-score	p, observed	p, adjusted	α, adjusted
sighted group, anatomical instruction: interaction between posture and congruency	14.77	<0.001	<0.001	0.025
sighted group, external instruction: interaction between posture and congruency	1.46	0.145	0.134	0.05
sighted group, external instruction, hands in same orientation, congruency effect	10.23	<0.001	<0.001	0.013
sighted group, anatomical instruction, hands in same orientation, congruency effect	8.34	<0.001	<0.001	0.017
sighted group, anatomical instruction, differently oriented hands, congruency effect	7.81	<0.001	<0.001	0.025
sighted group, external instruction, differently oriented hands, congruency effect	-7.28	<0.001	<0.001	0.05
blind group, external instruction: interaction between posture and congruency	4.92	<0.001	<0.001	0.025
blind group, anatomical instruction: interaction between posture and congruency	1.72	0.085	0.085	0.05
blind group, external instruction, hands in same orientation, congruency effect	3.75	<0.001	0.001	0.013
blind group, anatomical instruction, hands in same orientation, congruency effect	3.01	0.003	0.008	0.017
blind group, external instruction, differently oriented hands, congruency effect	-2.55	0.011	0.021	0.025
blind group, anatomical instruction, differently oriented hands, congruency effect	1.15	0.251	0.251	0.05

Table D. Post-hoc comparisons for direct comparison of performance in sighted and congenitally blind participant groups. Values in this table are Holm corrected (first row in each section) and uncorrected (second row). In addition, the corresponding adjusted α level is reported. Table 3 in the main text contains Holm corrected values. Values with p < 0.05 are set in bold font.

a, Interaction between Group and Congruency for each instruction and posture.

	anatomical instruction		external instruction	
	same posture	different posture	same posture	different posture
z-value	**3.65**	**4.81**	**2.99**	**-3.88**
p adjusted	**0.001**	**< 0.001**	**0.003**	**< 0.001**
p uncorrected	**< 0.001**	**< 0.001**	**0.003**	**< 0.001**
α adjusted	0.017	0.05	0.013	0.025

b, Comparison between groups for each combination of Instruction, Posture, and Congruency

		anatomical instruction		external instruction	
		same posture	different posture	same posture	different posture
congruent	z-value	-0.13	-0.29	0.73	2.40
	p adjusted	0.082	0. 082	0.082	0.082
	p uncorrected	0.895	0.774	0.465	**0.016**
	α adjusted	0.013	0.013	0.013	0.010
incongruent	z-value	**2.67**	**2.85**	**3.35**	-0.09
	p adjusted	**0.045**	**0.031**	**0.006**	0. 082
	Uncorrected	**0.008**	**0.004**	**< 0.001**	0.932
	α adjusted	0.008	0.007	0.006	0.013

References

Barr, D. J., Levy, R., Scheepers, C., & Tily, H. J. (2013). Random effects structure for confirmatory hypothesis testing: Keep it maximal. *Journal of Memory and Language*, *68*(3), 255–278. https://doi.org/10.1016/j.jml.2012.11.001

Bates, D., Kliegl, R., Vasishth, S., & Baayen, H. (2015). Parsimonious Mixed Models. *arXiv:1506.04967 [Stat]*. Retrieved from http://arxiv.org/abs/1506.04967

Box, G. E. P., & Cox, D. R. (1964). An Analysis of Transformations. *Journal of the Royal Statistical Society. Series B (Methodological)*, *26*(2), 211–252.

Holm, S. (1979). A Simple Sequentially Rejective Multiple Test Procedure. *Scandinavian Journal of Statistics*, *6*(2), 65–70.

Lenth, R. V., & Hervé, M. (2015). *lsmeans: Least-Squares Means*. Retrieved from http://CRAN.R-project.org/package=lsmeans

Pinheiro, J. C., & Bates, D. (2000). *Mixed-Effects Models in S and S-PLUS*. New York, NY: Springer.

Singmann, H., Bolker, B., & Westfall, J. (2015). *afex: Analysis of Factorial Experiments*. Retrieved from http://CRAN.R-project.org/package=afex
